# Confining Pressure Forecasting of Shield Tunnel Lining Based on GRU Model and RNN Model

**DOI:** 10.3390/s24030866

**Published:** 2024-01-29

**Authors:** Min Wang, Xiao-Wei Ye, Jin-Dian Jia, Xin-Hong Ying, Yang Ding, Di Zhang, Feng Sun

**Affiliations:** 1 Polytechnic Institute, Zhejiang University, Hangzhou 310058, China; 11912133@zju.edu.cn; 2 Department of Civil Engineering, Zhejiang University, Hangzhou 310058, China; cexwye@zju.edu.cn (X.-W.Y.); 22212018@zju.edu.cn (J.-D.J.); 22212019@zju.edu.cn (X.-H.Y.); 3 Department of Civil Engineering, Hangzhou City University, Hangzhou 310015, China; 4 China Railway Siyuan Survey and Design Group Co., Ltd., Wuhan 430063, China; zdiok@163.com (D.Z.); sunfeng5433@163.com (F.S.)

**Keywords:** shield tunnel, confining pressure, time series forecasting, Gate Recurrent Unit (GRU), recurrent neural network (RNN), multi-output recursive strategy

## Abstract

The confining pressure has a great effect on the internal force of the tunnel. During construction, the confining pressure which has a crucial impact on tunnel construction changes due to the variation of groundwater level and applied load. Therefore, the safety of tunnels must have the magnitude of confining pressure accurately estimated. In this study, a complete tunnel confining pressure time axis was obtained through high-frequency field monitoring, the data are segmented into a training set and a testing set. Using GRU and RNN models, a confining pressure prediction model was established, and the prediction results were analyzed. The results indicate that the GRU model has a fast-training speed and higher accuracy. On the other hand, the training speed of the RNN model is slow, with lower accuracy. The dynamic characteristics of soil pressure during tunnel construction require accurate prediction models to maintain the safety of the tunnel. The comparison between GRU and RNN models not only highlights the advantages of the GRU model but also emphasizes the necessity of balancing speed accuracy in tunnel construction confining pressure prediction modeling. This study is helpful in improving the understanding of soil pressure dynamics and developing effective prediction tools to promote safer and more reliable tunnel construction practices.

## 1. Introduction

The tunnel is generally equipped with comprehensive tunnel electromechanical facilities. However, when collecting tunnel monitoring data, the stability of the equipment and many factors related to the surrounding environment often result in data loss, affecting the integrity and accuracy of the data. When the latest data are missing, missing parts can be predicted based on the existing data. Therefore, the prediction of tunnel data is crucial for a better analysis of tunnel conditions and safety.

The confining pressure of the tunnel is the main source of the internal force of the tunnel. Excessive confining pressure changes will lead to the destruction of the tunnel segment, the increase in the gap at the interface between the segments, and the failure of the bolts between the connecting segments. Therefore, to ensure the safety of the tunnel, it is necessary to research the accurate calculation of the confining pressure of the shield tunnel. However, the pressure of the soil around the tunnel will change with factors [1], which makes it difficult to accurately calculate the confining pressure of the tunnel. Therefore, it is necessary to research the accurate calculation of the confining pressure of the shield tunnel. There have been many studies on the calculation method of tunnel structural confinement pressure. In terms of theoretical research, the limit analysis theory was first proposed by Soviet scholars. Initially, the theory was used to solve the ultimate bearing capacity of bar systems and concrete structures. After that, Drucker proposed the associated flow rule which is based on the premise of stable materials, and established a complete plastic limit analysis theory by combining the static field and velocity field with Prager w and Greenberg HJ et al. and used this theory to study the plane and space problems of ideal elastic–plastic materials. So far, the complete plastic limit analysis theory has been established [2,3]. Davis et al. [4] proposed four failure modes of circular tunnels shallowly buried in clay under undrained conditions and obtained the upper bound solution of the support reaction force required for tunnel instability under four conditions by combining the limit analysis theory. Then, the static allowable stress field was constructed to obtain the lower bound solution of the support reaction force. Takemura [5] proposed a failure mode utilizing the findings from centrifuge testing of shallow circular tunnels. This failure mode fully considers the soil’s shear strength anisotropy and the impact of burial depth on shear strength. On this basis, the derivation of the surrounding rock pressure expression is validated for feasibility in comparison with the test results. Lyu et al. [6] proposed two failure modes of layered soil double-hole tunnels, considering the different positions of the tunnel failure surface because the current tunnel specification is only applicable to the calculation of rock pressure around a double-hole tunnel in homogeneous soil. A calculation formula for the surrounding rock pressure is developed. The accuracy of the analysis method is confirmed by comparing the theoretical solution with numerical simulations and field measurements. Zhou et al. [7] proposed a failure mode of an ultra-shallow buried bias tunnel according to the actual engineering and derived the calculation formula for the surrounding rock pressure based on the limit equilibrium theory. The findings indicate that the vertical pressure on the tunnel primarily originates from the overlying soil’s gravitational force, and the lateral pressure on the tunnel is derived from the surrounding rock of the tunnel’s sidewall. Utilizing the limit analysis upper and lower bound theorems, Atkinson and Potts [8] studied the stability of shallow buried tunnels in cohesionless soil under plane-strain conditions and validated the precision of the theoretical solution through model tests. Leveraging the upper bound theorem of limit analysis and the Hoek–Brown criterion, Wang Atkinson [9] derived the analytical formulas of surrounding rock pressure of a layered, shallow tunnel under two-dimensional and three-dimensional failure modes and investigated the connection between surrounding rock pressure and tunnel dimensions as well as the mechanical properties of the surrounding rock. Tu et al. [10] constructed a failure mode observed in large-span shallow-buried tunnels within upper-soft and lower-hard rock strata and obtained the corresponding formula for calculating surrounding rock pressure utilizing the nonlinear Hoek–Brown failure criterion in conjunction with the upper bound theorem. By comparing the theoretical calculation for the pressure exerted by surrounding rock with the field measurement results, the correctness of the theoretical method was verified.

The model offers a controlled and efficient way to explore, understand, and test various phenomena. It can restore the parameter attributes of the actual soil and complex boundary constraints and monitor the development of stress and deformation during the experiment and analyze a series of problems such as surrounding rock stability according to the monitoring data results. The porosity, permeability, and saturation of soil can be obtained by electrical resistivity tomography [11], which can provide parameters for model tests. Schofield [12] completed two groups of soft soil tunnel excavation tests with a model ratio of 1/75 and 1/125 using a centrifuge independently developed by Cambridge. The centrifuge accurately restores the stress history of the soil, monitors the tunnel’s deformation during excavation, and assesses the surrounding rock pressure during excavation, with the obtained results broadly aligning with theoretical expectations. Lei et al. [13] studied the excavation process of tunnels with different surface inclination angles by model tests, highlighting the distribution and variation of pressure of the surrounding rock. The results show that the confining pressure at the excavation surface is the most obvious, and the confining pressure during excavation forms a cone shape, with the tunnel as the apex and the slope as the base. Xu et al. [14] considered the scenario where the tunnel intersects a fault fracture zone and adopted the model test, simulating the entire tunnel excavation process. The spatial distribution of surrounding rock pressure was obtained by the pre-buried confining pressure sensor. The experimental and numerical results revealed a similar distribution trend. Xu et al. [15] conducted model tests to investigate the distribution pattern of surrounding rock pressure when the tunnel traverses the gully in both backward and forward directions. The results show that during the excavation process, the maximum value of surrounding rock pressure appears at the vault or spandrel, and the minimum value appears at the arch foot. By studying the influence of the excavation direction and gully inclination angle on surrounding rock pressure, it is observed that the excavation direction significantly influences the distribution of surrounding rock pressure.

With the development of computer technology, the numerical method is widely used in the field of geotechnical engineering. Udomchai et al. [16] verified that the new revetment structure has sufficient internal and external failure safety factors through finite element simulation, and the results show that the structure is safe. Ding et al. [17] proposed a settlement-probability prediction model with a Bayesian emulator based on the Gaussian prior and verified the validity of the model by using the 1-year settlement monitoring data collected by a structural health monitoring system. Chen et al. [18] proposed a method for calculating the vertical Earth pressure of tunnel lining based on the results of the finite element model. Li et al. [19] proposed a general formula for estimating the vertical Earth pressure of the foundation and verified it using the finite element model. Based on the project of Metro Line 3 of Xi’an crossing ground fissures, Peng et al. [20] combined the discrete element method simulation with model tests, and the study investigated the surface deformation and support structure deformation. Jiang and Yin [21] studied the redistribution of surrounding rock stress and the variation law of surrounding rock pressure during shield tunnel construction in sandy soil layers. Ding et al. [22] compared the predicted settlement effects of different models and suggested that the GP model should be used to predict the tunnel settlement.

Currently, the main approach for recovering missing tunnel data is interpolation, which can be categorized into statistical interpolation and machine learning interpolation [23,24,25,26]. Although multiple imputation is more accurate than single imputation, it requires a significant amount of data and involves complex modeling [27]. Shen et al. [28] constructed Random Forest models and a BP neural network to predict tunnel accident states and durations. Shi et al. [29] precisely forecasted the highest surface settlement resulting from tunnel boring by utilizing a neural network. Suwansawa and Einstein [30] demonstrated the application of BP neural networks in Earth pressure balance shield tunneling based on a large amount of field measurement data. Zhou et al. [31] used Random Forest to predict surface settlement, and the prediction results of Random Forest were obtained by combining multiple embedded calculation results, resulting in high accuracy. Simeoni et al. [32] proposed a data fusion modeling approach for slope failure analysis and a reliability assessment method for testing data and results. Based on the LSTM, PSO and multi-output recursive strategy, Ye et al. [33] constructed a model to predict the 7-day history of confining pressure. Results show that the models have better prediction performance.

Recently, the rapid advancement of the geotechnical engineering field has been driven by the increasing utilization of machine learning techniques. Tunnel data prediction progress is very rapid. This study compares the performance differences between GRU and RNN machine learning methods in predicting missing tunnel data, aiming to find the optimal prediction model.

## 2. Machine Learning Models

### 2.1. Backpropagation Neural Network Model

**Figure 1:**
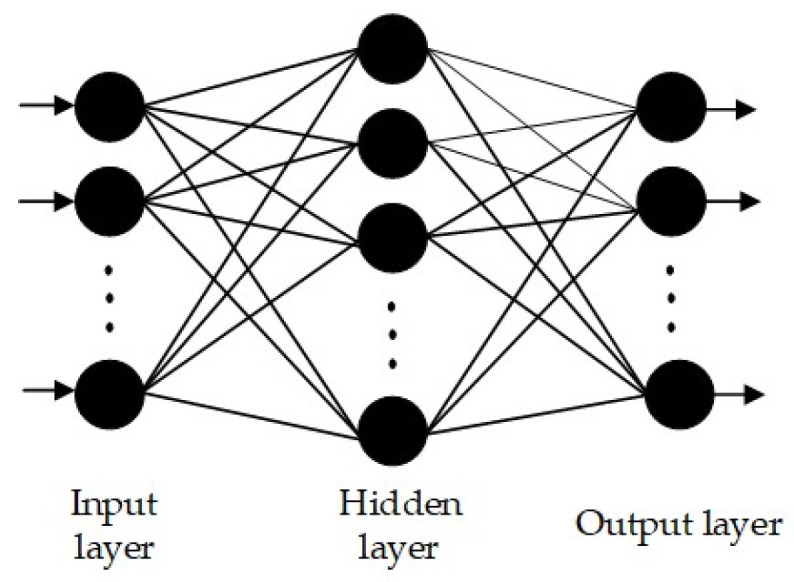
Structure diagram of BP neural network.

Among numerous neural network models, the backpropagation neural network (BPNN) is extensively used across diverse domains owing to its high precision and reliability [34]. A neural network typically consists of the following layers: the input layer, several hidden layers, and the output layer. Each layer contains a specific quantity of neurons. Adjacent layers of neurons are linked through weighted network connections, where each neuron receives input from all neurons in the preceding layer. The core of the BPNN lies in establishing a relationship between the output and input using an approximate mathematical model, achieved through an error feedback algorithm. The structure of the BPNN is depicted in Figure 1.

The learning process of the BP neural network comprises two parts: forward propagation of information and backward propagation of errors. The input information of the BPNN is transmitted from the input layer to the hidden layer. Subsequently, through an activation function, the neurons in the hidden layer transmit information to the output layer, resulting in the final output. The activation function enables nonlinear mapping of the information. If the error of the output is too big, error backpropagation occurs. The network weights of each layer will be changed to reduce the errors in the net. This process is repeated many times until the error meets the requirements. The basic idea of the backpropagation algorithm (BP) is to continuously reduce its learning error by moving the BP neural network in the downward direction of the gradient. In this algorithm, the mean square error of the BP network is represented as follows:

(1) $$E=\frac{1}{2}\sum_{k=1}^{l}{\left(d_{k}-O_{k}\right)}^{2}$$

where *l* is the total number of output neurons, $d_{k}$ is the expected output of the *k*-th output neuron and is the actual output of the *k*-th output neuron.

This expression can be represented as follows:

(2) $$E=\frac{1}{2}\sum_{k=1}^{l}{\left\{d_{k}-\left[\sum_{j=1}^{m}V_{ij}f\left(\sum_{i=1}^{n}W_{ij}X_{j}\right)\right]\right\}}^{2}$$

where *n* is the total number of input neurons, and *m* is the total number of hidden layer neurons.

The expression for the weight increment of the connection between the *i*-th input layer neuron and the *j*-th hidden layer neuron can be expressed as follows:

(3) $$\Delta W_{ij}=-\eta \frac{\partial E}{\partial W_{ij}}$$

(4) $$\Delta V_{ij}=-\eta \frac{\partial E}{\partial V_{ij}}$$

where *η* is the learning rate.

So, $\Delta W_{ij}$ and $\Delta V_{ij}$ are expressed as follows:

(5) $$\Delta W_{ij}=-\eta X_{i}\left[\sum_{k=1}^{l}\left(d_{k}-O_{k}\right)f^{\prime }\left(net_{k}\right)V_{jk}\right]f^{\prime }\left(net_{j}\right)$$

(6) $$\Delta V_{ij}=-\eta Y_{j}\left[d_{k}-O_{k}\right]f^{\prime }\left(net_{k}\right)$$

where is the output of the *k*-th output neuron, $net_{j}$ is the output of the *j*-th hidden layer neuron, and *η* is the learning rate.

### 2.2. Random Forest

**Figure 2:**
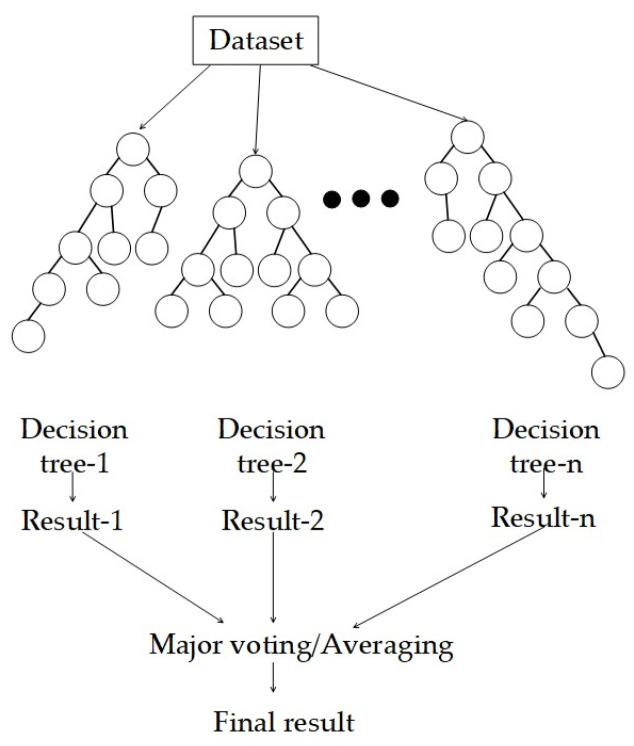
Random Forest.

The decision tree algorithm is a classical data mining technique that essentially involves recursively classifying data using a series of rules, as shown in Figure 2. However, single decision trees suffer from limitations such as low accuracy and susceptibility to overfitting. To address these issues, ensemble learning has become a prominent research focus in machine learning. In 2001, Breiman combined his Bagging theory [35] with the CART decision tree algorithm and Ho’s Random Subspace Method to propose a non-parametric classification and regression algorithm called Random Forest (RF) [36]. Random Forest has gained significant attention and popularity in the machine-learning community due to its ability to mitigate overfitting and improve accuracy by integrating multiple algorithms.

The basic idea of Random Forest is as follows: Firstly, the bootstrap resampling technique is used to select samples with replacement from the initial training dataset D, generating a training set. Many decision trees are built based on the bootstrap sample set, forming a Random Forest. Finally, for a given input sample for classification or regression, the Random Forest combines the output results of each decision tree using either a simple majority voting or averaging of the outputs to determine the final prediction result.

In recent years, extensive theoretical research and empirical validation have demonstrated that Random Forest possesses the ability to analyze complex relationships in data. It can effectively handle high-dimensional data by performing feature selection and dimensionality reduction through variable importance ranking. Random Forest also exhibits robustness against noise and missing data, and it offers several advantages such as high prediction accuracy and reduced risk of overfitting.

### 2.3. Support Vector Machines

**Figure 3:**
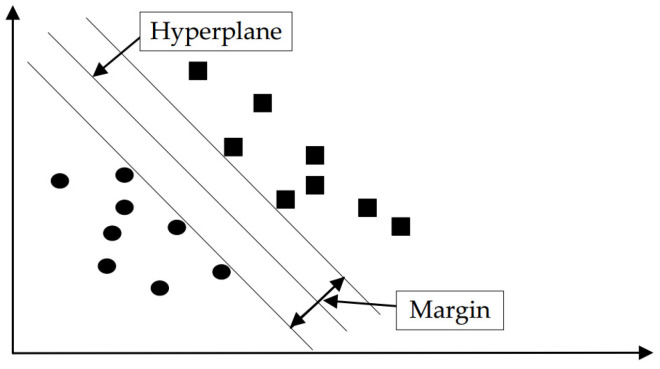
Support vector machines.

The SVM model is developed based on statistical principles, as shown in Figure 3 [37,38]. It establishes a hyperplane that minimizes the loss of training samples and achieves the best fit. The basic concept of SVM is to map data samples to high-size functionality spaces, where samples from different classes can be separated by a hyperplane. In the feature space, these closest samples to the hyperplane determine the position and orientation of the hyperplane. The classification process of SVM can be achieved by computing the distance from samples to the hyperplane. For new test samples, their class membership can be determined by calculating their distance to the hyperplane.

The essence of the SVM is to solve an optimization problem to determine the parameters of the hyperplane. Common optimization problems include hard margin maximization and soft margin maximization. The objective of hard margin maximization is to find a hyperplane that correctly classifies all samples while maximizing the distance between the support vectors and the hyperplane. Soft margin maximization takes the noise into consideration and outliers in the sample data, permitting a margin of misclassified samples to enhance the robustness of the model.

The SVM algorithm exhibits good generalization ability and robustness, and it has achieved favorable results in many practical applications. However, for large-scale datasets and high-dimensional feature spaces, SVM’s computational complexity is high, requiring significant time and computational resources. Therefore, in practical applications, it is necessary to choose appropriate algorithms and optimization methods to improve efficiency based on specific circumstances.

### 2.4. K-Nearest Neighbors

**Figure 4:**
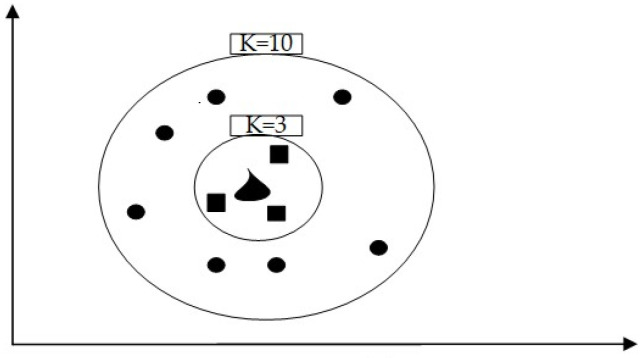
K-nearest neighbors.

The KNN is an algorithm used for both classification and regression that measures the distances between different samples and selects the K-nearest neighbors for classification, as shown in Figure 4 [39,40]. The KNN algorithm is based on distance measurement between instances and predicts the category or the value of a sample by finding the K-closest training samples. The fundamental procedure of the KNN algorithm is outlined below: (1) Prepare data set: collect features and label data of the training samples to build the KNN model. (2) Choose K’s value: Determine the value of K which represents the count of nearest neighbors to consider during prediction. The choice of K value is usually based on techniques like cross-validation or other model selection methods. (3) Compute distances: for a new sample, calculate the distances between the new sample and each sample in the training set using an appropriate distance measure. (4) Choosing the closest neighbors: using distance measures to select the K training samples closest to the new sample. (5) Make predictions: For classification problems, use a voting mechanism to determine the class of the new sample. That is, select the most frequently occurring class among the K-nearest neighbors as the prediction result. For regression problems, the predicted value can be determined by averaging the values of the K-nearest neighbors. KNN is a simple yet effective method that relies on distance measurement and neighbor selection for classification and regression tasks.

### 2.5. Recurrent Neural Network

**Figure 5:**
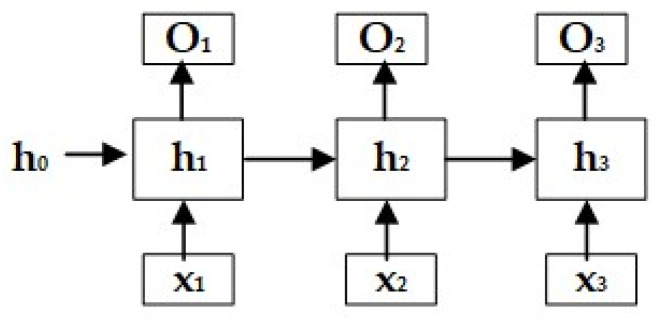
Recurrent neural network.

Recurrent neural network is a type of deep neural network designed to recognize and handle time series data, as shown in Figure 5 [41]. In a BP neural network, the signal data are forward propagated in a BP neural network, that is, the data can only be transmitted from the upper layer to the next layer, and in the process of processing, the data at each moment are relatively independent. The recurrent neural network introduces the concept of time series into the neural network, that is, the input value obtained by a layer of neurons at time *t* includes not only the output weighted sum of the previous layer but also the output value of the layer itself at (*t* − 1) or (*t* + 1).

As a type of supervised learning model, the training objective of RNN is to minimize the objective function. To achieve this training goal, RNN uses the Backpropagation Through Time (BPTT) algorithm [42] to compute the extended gradients on the model, thereby optimizing the network model’s parameters. When extended, RNN can be viewed as a multi-layer feedforward neural network with shared parameters. The length of the time series (the number of time nodes) corresponds to the number of expanded layers. Too many layers will not only slow down the training speed of the model but also cause the problem of gradient ‘explosion’ and gradient ‘disappearance’ when applying the time-lapse backpropagation algorithm, resulting in RNN being unable to obtain long-term dependent information, thus losing the ability to use long-distance historical information.

Recurrent neural networks have two types: the Jordan network and the Elman network. Compared to the Jordan network, the Elman network has a simpler structure and higher operational efficiency. Therefore, the mainstream RNN architecture is currently the Elman network. RNN is also the default Elman network. RNN is similar to the BP neural network. Its core structure also consists of the input layer, hidden layer, and output layer, connected by weighted connections between layers. The core algorithm is also able to find the gradient of each parameter. The biggest difference between them is that RNN realizes the weight connection of all neurons in the same layer. In the standard RNN, the output of a node in the hidden layer at *t* time not only enters the output layer at *t* time but also becomes the input of the hidden layer at time *t* + 1. In this way, RNN realizes the temporality within the network, which gives it great advantages in processing time series data.

### 2.6. Gate Recurrent Unit

**Figure 6:**
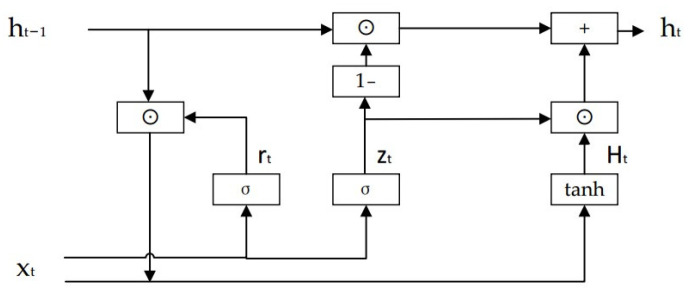
Gate Recurrent Unit.

GRU and LSTM are common RNN improvement methods, as shown in Figure 6. Although LSTM solves the long-term memory problem, the network structure is relatively complex. Especially when LSTM units are connected in series, it is equivalent to introducing a multi-layer feedforward neural network, which is easy to cause slow training and non-convergence of results. Cho [43] proposed the Gated Recurrent Unit (GRU) neural network. The GRU was introduced as a more streamlined and efficient alternative to LSTM networks, addressing some of the complexities associated with LSTM. The input gate and forgetting gate of LSTM are merged into the update gate to form the gating structure of the update gate and the reset gate, and the corresponding weight parameters are reduced. The basic structure is shown in Figure 6.

The working principle of the GRU unit is as follows:

(1) Update gate: The output $h_{t-1}$ at time *t* − 1 and the input $x_{t}$ at time *t* are input into the sigmoid, the output is $z_{t}$, and the value is [0, 1]. Its calculation formula is as follows:
  (7) $$z_{t}=\sigma \left(W_{z}\cdot \left[h_{t-1},x_{t}\right]\right)$$
  where $W_{z}$ is the weight matrix.
(2) Reset gate: input $h_{t-1}$ and $x_{t}$ into the sigmoid function, the output $r_{t}$ is a value between [0, 1]. Its calculation formula is as follows:
  (8) $$r_{t}=\sigma \left(W_{r}\cdot \left[h_{t-1},x_{t}\right]\right)$$
  where $W_{r}$ is the weight matrix.
(3) Candidate hidden state: $r_{t}$ and $h_{t-1}$ are multiplied, and the result and $x_{t}$ are input into tanh. The output is the candidate hidden state $H_{t}$ at time *t*, and the value is [−1, 1]. Its calculation formula is as follows:
  (9) $$H_{t}=\tanh\left(W_{\tilde{h}}\cdot \left[r_{t}*h_{t-1},x_{t}\right]\right)$$

When $r_{t}$ = 0, the output of the reset gate is 0, then the product of $r_{t}$ and $h_{t-1}$ is 0, which means that the information at *t* − 1 is useless for the current state. In other words, the current state is only related to the current input data, which resets the hidden layer state. It is precisely because of this that the reset gate is conducive to capturing short-term dependencies.

(4) Hidden state: the output $z_{t}$ of the update gate is linearly combined with the state $h_{t-1}$ at time *t* − 1 and the candidate state $H_{t}$ at time *t*. Its calculation formula is as follows:
  (10) $$h_{t}=\left(1-z_{t}\right)*h_{t-1}+z_{t}*H_{t}$$

It can be seen from the above formula that when $z_{t}$ = 0, then 1 − $z_{t}$ = 1, and the hidden state of the previous moment is completely retained. At this time, if there is a long-term dependence, the hidden information can also be retained. It is precisely because of this that GRU can capture a long-term dependence, which is also the most important part of GRU. Therefore, the update gate helps to capture long-term dependencies.

## 3. Geological and Geotechnical Conditions

The river-crossing section of a tunnel project is constructed using the shield method. The length of the tunnel section is about 1254 m, the minimum curve radius is 1500 m, and the thickness of the overburdened soil in the middle section of the river is 9.7–13 m. Where the tunnel passes through, there is a lot of sand, round gravel, pebble soil, etc., among which the sandy pebble stratum that mainly passes through has the characteristics of a large inter-particle gap, low cohesion, and high sensitivity. In addition, the shield tunnel passes through the ultra-shallow overburden section at the bottom of the river (the minimum overburden thickness is 9.7 m, less than 0.7 D), with high water pressure and strong permeability. In the process of shield construction, the tunnel segment will float up under the action of various loads such as jack thrust, grouting pressure, slurry buoyancy, and stratum stress. The uneven floating will cause the segment to stagger, resulting in the initial internal force of the shield tunnel. This process involves the interaction of soil-segment-jack thrust. At the same time, it is affected by the construction sequence (tunneling attitude, assembly process, shield tail grouting). Theoretical analysis and numerical simulations are challenging to accurately reflect the stress state of the shield tunnel segments during this phase.

On the other hand, the tunnel is a large-diameter tunnel crossing a river jointly built with a highway and railway During the operation period, the long-term complex traffic dynamic cyclic load (subway train load, vehicle load) and the change in river water level will distribute internal and external loads on the tunnel, causing the longitudinal uneven deformation of the tunnel structure, and then generate stress concentration in the weak part of the tunnel structure. It will induce tunnel structure disease and affect service safety. Due to the failure to recover the tunnel monitoring data in time after the loss, there are major challenges in the efficient diagnosis of structural diseases and accurate evaluation of service performance. Engineering accidents caused by a failure to maintain the tunnel occur from time to time.

## 4. Monitoring Data and the Preprocessing Method

### 4.1. The Monitoring System of the Tunnel

In traditional tunnel monitoring systems, most observations and data measurements are primarily conducted manually, which will use plenty of human and material resources during construction and operation. In addition, its security is relatively low. The use of the Internet of Things system can not only improve the detection frequency, and save time and labor, but also provide accurate and timely changes in the envelope structure, timely correction of parameters and technology, and enough support for construction.

**Figure 7:**
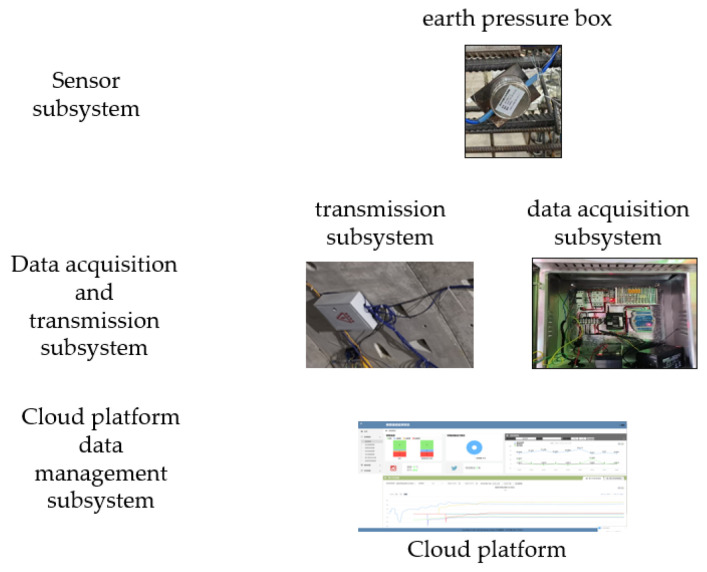
Monitoring system.

**Figure 8:**
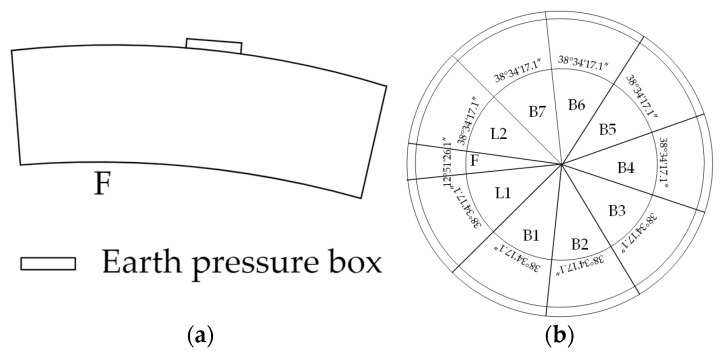
Sensor placement (**a**) the location of the Earth pressure box; (**b**) the location of the F block.

**Figure 9:**
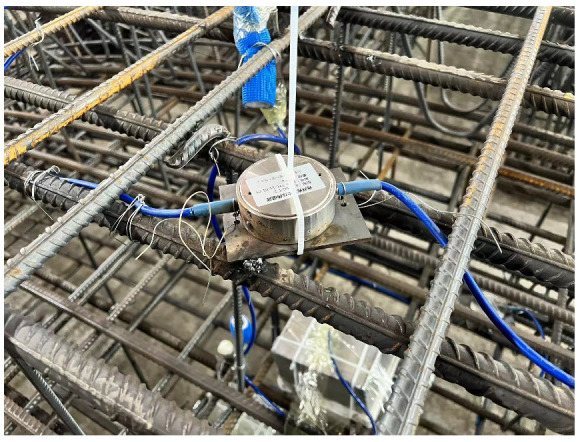
Earth pressure box.

The monitoring system of the tunnel is shown as Figure 7. There are several monitoring points in the ring, as shown in Figure 8. Fiber Bragg grating sensors are interconnected using optical cables. To make the data quality better, both ends of the optical cable are connected to the concealed box. After the casting and curing of the segments are completed, the concrete covering the Earth pressure box is chiseled off, as shown in Figure 9. Then, the handheld demodulator is used to collect the wavelength and frequency readings of the sensor, which are compared with the preset values to verify the reliability of the sensor during the monitoring process. The Earth pressure box is mainly used to monitor the magnitude of soil pressure. Due to the long monitoring period and the need for strong anti-interference and stability, an Earth pressure box based on a vibrating-wire is adopted. There are also many challenges in monitoring soil pressure: (1) sensors may be damaged during transportation and placement; and (2) during the construction process, factors such as power outages and electromagnetic interference increase the difficulty of on-site monitoring.

### 4.2. Time Series Pruning via Equidistant Sampling

**Figure 10:**
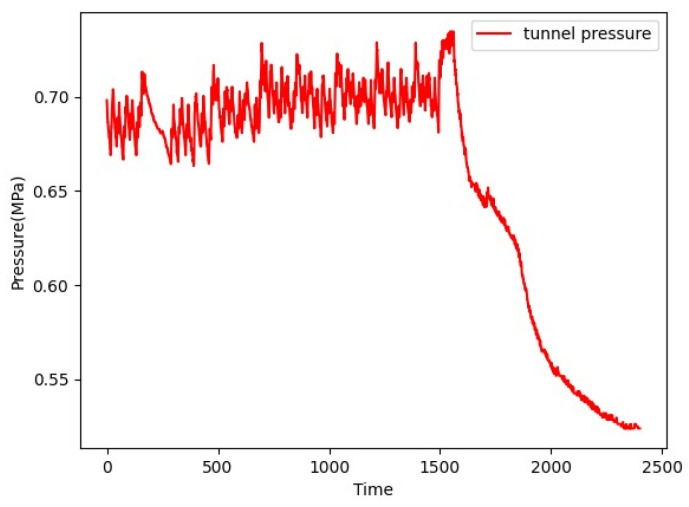
Monitoring data.

The original data come from a pressure sensor on a ring, as shown in Figure 10. The sampling rate is 1 S/s. The interval is 1 s, and the monitoring period is 40 min. The multi-step prediction of 4 min is to be achieved. Using raw data directly, 240 points should be predicted. The embedding size should be larger than forecasting horizon to avoid a sharp decline in prediction performance. 

### 4.3. Data Partitioning for Training and Testing

The forecasting horizon should be smaller than the embedding size to avoid a sharp decline in prediction performance. The original data come from a pressure sensor on a ring. The monitoring period is one second, the monitoring time is 40 min, and the multi-step prediction takes 4 min is to be achieved. Using raw data directly, 240 points need to be predicted. Since the forecasting horizon should be less than the embedded size, machine learning can be used for prediction. The training set and the test set are divided based on the chronological sequence. The first 90% of the data are selected as the training set, and the last 10% of the data are selected as the test set [44].

### 4.4. GRU Model and RNN Model

**Figure 11:**
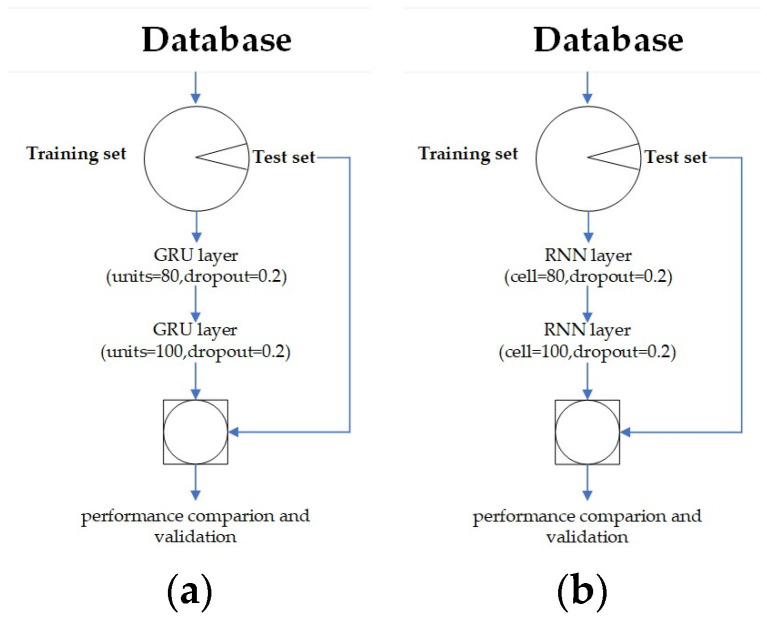
Research flowchart. (**a**) GRU; (**b**) RNN.

The monitoring data are time series data, the first 90% are selected as the training set, and the last 10% are selected as the test set. The data are normalized to the [0, 1] interval by a function. Each continuous 60 value is selected as a group, and the value of the next moment is predicted by the first 60 values. The GRU model has two GRU layers, the first layer has 100 units, and the dropout of the first layer is 0.2. The second layer has 80 units, and the dropout of the second layer is 0.2. The RNN model has two RNN layers. The first layer has 100 cells, and the dropout of the first layer is 0.2. There are 80 cells in the second layer, and the dropout of the second layer is 0.2. The research flowchart of the GRU and the RNN is shown in Figure 11.

## 5. Prediction of Confining Pressure Based on GRU Model and RNN Model

### 5.1. Model-Evaluation Index

To comprehensively evaluate the accuracy of the model, mean absolute percentage error (*MAPE*) [45], mean absolute error (*MAE*), the regression coefficient of determination *R*^2^, and root-mean-squared error (*RMSE*) are usually used to evaluate the results.

(11) $$MAPE=\frac{1}{n}\sum_{i=1}^{n}\frac{\left|{\hat{x}}_{i}-x_{i}\right|}{x_{i}}$$

(12) $$R^{2}=1-\left[\sum_{i=1}^{n}{\left({\hat{x}}_{l}-x_{i}\right)}^{2}/\sum_{i=1}^{n}{\left({\bar{x}}_{l}-x_{i}\right)}^{2}\right]$$

(13) $$MAE=\frac{1}{n}\sum_{i=1}^{n}\left|{\hat{x}}_{i}-x_{i}\right|$$

(14) $$RMSE=\sqrt{\frac{1}{n}\sum_{i=1}^{n}{\left({\hat{x}}_{l}-x_{i}\right)}^{2}}$$

where ${\hat{x}}_{l}$ is the predicted value; $x_{i}$ is the raw value; n is the length of the dataset; and ${\bar{x}}_{l}$ is the average of raw data.

### 5.2. Generalization Ability of Model

The training of the model is not simply to let it remember the training samples that have been learned. It is mainly through the learning of the training samples to find and restore the inherent regularity of the environment itself hidden in the training samples, so that the test or working samples can be given the correct output. To achieve this goal, it is necessary to study the main factors affecting the model’s ability to promote, such as the model structure, training methods, design, complexity of performance indicator functions, the quantity and quality of the training samples, the initial weight, and so on.

This paper briefly analyzes the generalization capability of the model and its relationship with the network structure, driving methods, and functional design of the performance indicator. The generalization capacity of the model is closely related to the structure of the network. If the structural parameters of the model are much smaller than those of the training sample, it can reduce the possibility of overfitting, which helps to improve the generalization capacity of the network. On the contrary, if there are too many training samples, the probability of learning system noise will increase. Suppose the network is trained by using the commonly used performance index of minimum mean-square error, and the error in the training set is excessively minimized. In that case, the model will remember some noise or individual special cases, and an over-fitting phenomenon will occur. The real system law will not be learned, thus reducing the generalization ability of the network.

It is also effective to adopt a suitable model training method to improve its generalization ability. If the network is trained by training methods such as the regularization method and optimal stopping training method, the generalization ability of the model can be improved.

### 5.3. The Prediction Results of the Model

#### 5.3.1. Overfitting

Overfitting means that the model makes the hypothesis too strict to obtain the consistency hypothesis. The model does not learn the overall distribution of the data, but it learns the expected output of each data point and relies too much on some local features in the data. In general, the performance of the overfitting problem is very perfect when the training effect can be performed, but the performance of the model on the unknown data will be poor after the training is completed. In general, the main reason for overfitting is that the training data are too small or the model is too complex.

With the deepening of related research, for the over-fitting problem, the commonly used solutions are as follows:

1. Data Augmentation: Most of the overfitting problems are caused by too little training data. Common data augmentation schemes include collecting more data samples that are independent and identically distributed or approximately independent and identically distributed with existing data; taking more data samples by adding random noise to the original data and expanding the data set. Data augmentation can effectively reduce the probability of overfitting.
2. Regularization: Regularization refers to the learning of constrained models to reduce the process of overfitting. L1 and L2 regularization add some restrictions to some parameters in the loss function by the penalty term. For example, the penalty term of L1 regularization minimizes the absolute value of the weight, and the penalty term of L2 regularization minimizes the square value of the weight.
3. Dropout [46]: During training, some neurons and neural connections can be randomly ignored and trained once with an incomplete neural network. At the second time, some others are randomly ignored, so that each prediction result will not depend on a certain part of the specific neurons, so that the neural network does not have the opportunity to rely too much on the training data.
4. Early Stop: This method mainly stops the training of the model before the iterative convergence of the training data set to prevent the occurrence of over-fitting. The specific method is to use a part of the training data set as the verification set. After each training selection process, the loss of the verification set is calculated, and the training is stopped when the loss is no longer increased. By this means, the weakening of the generalization ability of the model caused by excessive training is avoided.

#### 5.3.2. The Prediction Result

Since the early stop method was proposed, several stopping criteria have been proposed. One of the stopping criteria is only based on the change of verification error. The training will be stopped when the accuracy on the validation declines between several generations. This paper will determine the stopping epoch by this stopping criterion.

**Figure 12:**
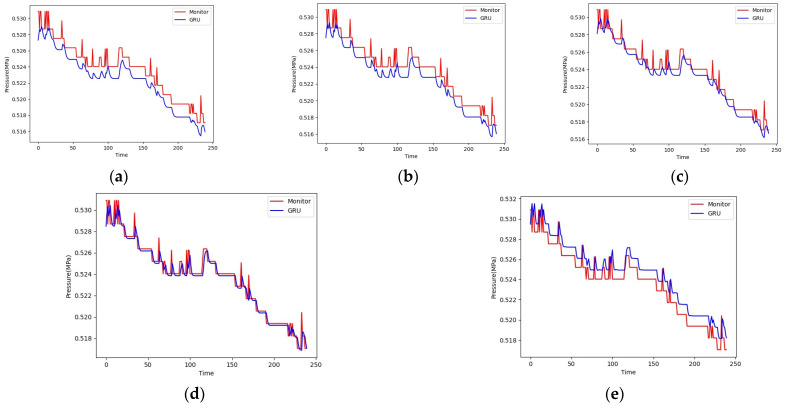
The prediction results of the GRU model for (**a**) epoch = 25; (**b**) epoch = 50; (**c**) epoch = 75; (**d**) epoch = 100; (**e**) epoch = 125.

The prediction curve and actual curve of the GRU model on the test set are shown in Figure 12. When training 100 epochs, the model has the highest accuracy on the test set. When training 125 epochs, the gap between the model and the true value becomes larger, the phenomenon of overfitting occurs, and the accuracy of the model decreases. The prediction results of the GRU model are relatively stable, and the results are very close to the true value.

**Figure 13:**
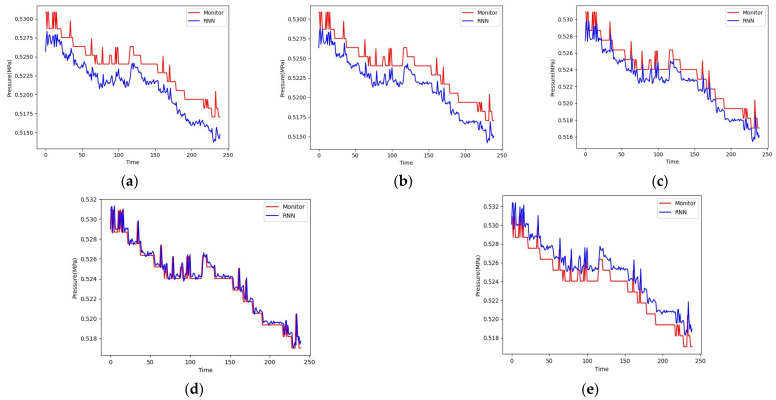
The prediction results of the RNN model for (**a**) epoch = 50; (**b**) epoch = 100; (**c**) epoch = 150; (**d**) epoch = 200; (**e**) epoch = 225.

The prediction curve and actual curve of the RNN model on the test set are shown in Figure 13. When training 200 epochs, the model has the highest accuracy on the test set. The predicted value is closer to the true value than the predicted value of GRU, but the model prediction results change greatly.

#### 5.3.3. Comparison of Model Prediction Effects

**Figure 14:**
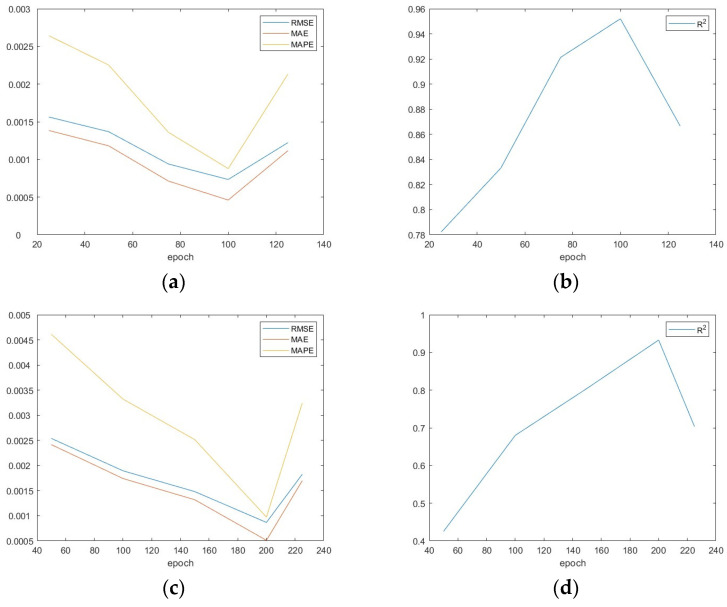
The model-evaluation index; (**a**) the MAPE MAE and RMSE of GRU; (**b**) the R^2^ of GRU; (**c**) the MAPE MAE and RMSE of RNN; (**d**) the R^2^ of RNN.

A multi-step prediction model based on GRU and a multi-step prediction model based on RNN are established, and the established models are compared. The RMSE, MAE, MAPE, and R^2^ of the GRU model and RNN model are shown in Figure 14. It is observed that RMSE, MAE, MAPE, and R^2^ in GRU model are 0.00073376 MPa, 0.00046113 MPa, 0.00087849, and 0.95201007, while RMSE, MAE, MAPE, and R^2^ in RNN model are 0.00086733 MPa, 0.0005112 MPa, 0.00097375, and 0.93294851. So, the GEU model performs better in predicting tunnel confining pressure.

## 6. Conclusions

This paper mainly completes the following work: (1) through on-site monitoring, the confining pressure of the shield tunnel construction period is described comprehensively; (2) the tunnel confining pressure is predicted by the GRU and RNN; and (3) comparing the predictive performance between two models. Based on the above work, the following conclusions can be drawn as follows:

1. By comparing the MAPE, MAE, RMSE, and R^2^ of the GRU model and RNN model, the training speed of the GRU model is faster, and the error between predicted results and actual values is small. The training speed of the RNN model is slow, and the error between predicted results and actual values is large.
2. The results indicate that the epoch of the model affects its effect. Therefore, the appropriate epoch should be selected for the model to ensure the prediction effect of the model. In general, the epoch difference of different models will be very large, and the Early Stop method can be used to find the appropriate epoch.
3. Generally speaking, the more complex the model is, the higher the prediction accuracy will be, but the higher the cost of training will be. In practical engineering, the target accuracy can be determined first, and then, the hyper-parameters can be selected for training to ensure that the training cost is the lowest under the premise that the model accuracy meets the requirements.

## Data Availability

Data are contained within the article.
